# Lung- and diaphragm-protective strategies in acute respiratory failure: an in silico trial

**DOI:** 10.1186/s40635-024-00606-x

**Published:** 2024-02-28

**Authors:** Damian Ratano, Binghao Zhang, Jose Dianti, Dimitrios Georgopoulos, Laurent J. Brochard, Timothy C. Y. Chan, Ewan C. Goligher

**Affiliations:** 1 Interdepartmental Division of Critical Care Medicine, University of Toronto, Toronto General Hospital, 585 University Ave, 9-MaRS-9024, Toronto, ON M5G 2N2 Canada; 2 Intensive Care and Burn Unit, Lausanne University Hospital (CHUV), Lausanne, Switzerland; 3 Department of Mechanical and Industrial Engineering, University of Toronto, Toronto, Canada; 4 Department of Intensive Care Medicine, University Hospital of Heraklion, University of Crete, Heraklion, Greece; 5 Division of Respirology, Department of Medicine, University Health Network, Toronto, Canada; 6 Toronto General Hospital Research Institute, University Health Network, Toronto, Canada; 7 Department of Physiology, University of Toronto, Toronto, Canada

**Keywords:** Acute respiratory failure, Protective ventilation, Extracorporeal CO_2_ removal, Diaphragm, In silico trial

## Abstract

**Background:**

Lung- and diaphragm-protective (LDP) ventilation may prevent diaphragm atrophy and patient self-inflicted lung injury in acute respiratory failure, but feasibility is uncertain. The objectives of this study were to estimate the proportion of patients achieving LDP targets in different modes of ventilation, and to identify predictors of need for extracorporeal carbon dioxide removal (ECCO_2_R) to achieve LDP targets.

**Methods:**

An in silico clinical trial was conducted using a previously published mathematical model of patient–ventilator interaction in a simulated patient population (*n* = 5000) with clinically relevant physiological characteristics. Ventilation and sedation were titrated according to a pre-defined algorithm in pressure support ventilation (PSV) and proportional assist ventilation (PAV+) modes, with or without adjunctive ECCO_2_R, and using ECCO_2_R alone (without ventilation or sedation). Random forest modelling was employed to identify patient-level factors associated with achieving targets.

**Results:**

After titration, the proportion of patients achieving targets was lower in PAV+ vs. PSV (37% vs. 43%, odds ratio 0.78, 95% CI 0.73–0.85). Adjunctive ECCO_2_R substantially increased the probability of achieving targets in both PSV and PAV+ (85% vs. 84%). ECCO_2_R alone without ventilation or sedation achieved LDP targets in 9%. The main determinants of success without ECCO_2_R were lung compliance, ventilatory ratio, and strong ion difference. In silico trial results corresponded closely with the results obtained in a clinical trial of the LDP titration algorithm (*n* = 30).

**Conclusions:**

In this in silico trial, many patients required ECCO_2_R in combination with mechanical ventilation and sedation to achieve LDP targets. ECCO_2_R increased the probability of achieving LDP targets in patients with intermediate degrees of derangement in elastance and ventilatory ratio.

**Supplementary Information:**

The online version contains supplementary material available at 10.1186/s40635-024-00606-x.

## Introduction

Mechanical ventilation is a life-saving intervention for patients with acute respiratory failure. However, it can injure both the lung and the diaphragm, and these injuries are associated with significant long-term morbidity and mortality [1–7]. Whilst the benefit of lung-protective mechanical ventilation is well-established [8, 9], the idea of diaphragm-protective ventilation has only recently been proposed and its benefit is as yet unproven [10]. In theory, lung- and diaphragm-protective (LDP) ventilation conditions could be achieved by targeting an optimal level of respiratory effort whilst maintaining safe limits on lung-distending pressure. Specifically, an international consensus framework proposed the following LDP targets: dynamic transpulmonary driving pressure (lung-distending pressure, ΔP_L,dyn_) < 15 cm H_2_O, an esophageal pressure swing (respiratory effort, ΔPes) of − 3 to − 8 cm H_2_O, and a pH > 7.25 [11]. Several areas of uncertainty need to be addressed before full-scale clinical trials of the LDP approach are undertaken. First, the feasibility of achieving these targets in the clinical setting has not been established. Second, the potential benefit of proportional assistance modes of mechanical ventilation to facilitate the LDP strategy is uncertain. PAV+ is theoretically superior to PSV for achieving LDP targets because in PAV+ the pressure delivered by the ventilator depends on the patient’s respiratory effort and the total distending pressure is subject to reflex and chemoreceptive feedback systems [12, 13]. Third, although applying extracorporeal CO_2_ removal (ECCO_2_R) to unload ventilatory demands may enable more direct control of respiratory effort [14–16], its utility to achieve LDP targets has not been evaluated.

We recently described a physiology-based mathematical model [17] to simulate the effect of modifying ventilation and sedation on acid–base homeostasis, respiratory effort, and lung-distending pressure. In the present study, this model was deployed as a digital simulator to conduct an in silico clinical trial of a pre-defined algorithm, tested in a previously published clinical study, for titrating ventilator support and sedation to achieve LDP targets [18].

The main objective of this study was to compare the rate of success in achieving LDP targets using the ventilation/sedation titration algorithm in PSV vs. PAV+ + modes, with or without adjunctive ECCO_2_R. We also aimed to assess the probability of achieving LDP targets by ECCO_2_R alone (without sedation and ventilatory support, hereafter termed ‘awake ECCO_2_R’). To assess the validity of the physiology-based model simulations, we compared the results of the in silico trial to the observed results of a pilot clinical trial testing the LDP ventilation/sedation algorithm in patients with acute hypoxemic respiratory failure [18]. A secondary objective of this study was to explore which readily available clinical variables could predict whether LDP targets could be achieved with or without adjunctive ECCO_2_R and to derive a score that could be prospectively tested in future studies.

## Methods

**Fig. 1:**
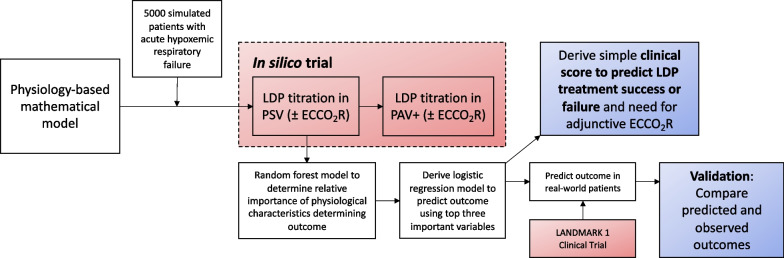
Research approach. The in silico trial was implemented by applying the physiology-based mathematical model to predict the effect of titrating ventilatory support (in PSV and PAV+ modes) and sedation according to a predefined algorithm on LDP targets in a simulated population of patients with acute hypoxemic respiratory failure. To assess the face validity of the in silico trial results, the predicted probability of achieving LDP targets (computed by a statistical model derived from the in silico trial) was compared to the observed outcome in a real-world clinical trial of the titration procedure

The overall approach to simulations and modelling in this study is summarized in Fig. 1.

### Physiological model

We recently described [17] a mathematical model of control of breathing during mechanical ventilation based on known physiological relationships governing respiratory mechanics, control of breathing [19, 20], acid–base homeostasis (using the Stewart approach) [21], ventilation, and pharmacokinetic and pharmacodynamic models of the effect of propofol on respiratory effort [22, 23]. The model predicts ΔPes, ΔP_L,dyn_, and pH in response to varying inspiratory support or propofol infusion rate in two different modes of assisted ventilation, pressure support ventilation (PSV) and proportional assistance ventilation with load-adjustable gain factors (PAV+). The model can also predict the effect of varying levels of ECCO_2_R on ΔPes, ΔP_L,dyn_, and pH.

### Simulated patient population for the in silico clinical trial

For this trial, an in silico population of 5000 patients was generated by randomly selecting values  from distributions of each of the parameters required for the physiological model computations (Additional file 1: Table S1). The parameter distributions were derived from previously published clinical studies on acute respiratory failure [2, 8, 9, 24–31]. The same in silico population was used for each simulated intervention.

### In silico titration of ventilation, sedation, and extracorporeal CO_2_ removal in PSV mode

Computational details for the simulation method based on the physiological model are detailed in Additional file 1: Online Supplement (section: Simulation Procedure). At baseline, patients were ventilated in PSV mode with an initial inspiratory pressure support of 10 cm H_2_O and an initial propofol infusion rate of 20 mcg/kg/min. Based on these settings, the physiological model computed baseline pH, ΔPes, and ΔP_L,dyn_ for each simulated patient. Patients who met the LDP targets at baseline were classified as “primary success”. In patients not meeting LDP targets at baseline, the pressure support level and propofol infusion rate were titrated according to the pre-defined algorithm (Additional file 1: Figure S1). After each titration of either the pressure support level or the propofol infusion rate, the physiological model re-computed pH, ΔPes, and ΔP_L,dyn_. If all three LDP targets were achieved at any time after a step of titration, the patient was classified as a “treatment success.” If the LDP targets were not reached after 20 iterations of the algorithm, a patient was classified as having “treatment failure.”

In the case of treatment failure, adjunctive ECCO_2_R was simulated at progressively escalating CO_2_ removal rates (i.e. decreasing the apparent rate of CO_2_ production in the model) up to a maximum of 90% of VCO_2_. At each ECCO_2_R level, the titration algorithm was re-run. If LDPV targets were achieved, the titration was halted.

### In silico titration of ventilation, sedation, and extracorporeal CO_2_ removal in PAV+ mode

The same simulation procedure was performed in the same simulated patient population using PAV+ mode with an initial PAV+ assist level of 50% and an initial propofol infusion rate of 20 mcg/kg/min. In patients who did not meet LDP targets at baseline under these settings, PAV+ assist level and propofol infusion rate were titrated according to the pre-defined algorithm as above. In patients who did not achieve LDP targets after the same titration procedure performed in PAV+, simulated ECCO_2_R was applied as above.

Because the intervention was applied in PSV and PAV+ in the identical simulated patient population, and because this simulated trial design is not susceptible to wash-out or order effects, no randomization was performed in the in silico trial design.

### In silico evaluation of ECCO_2_R without mechanical ventilation or sedation

To simulate the possibility of achieving LDP targets under ‘awake ECCO2R’ without using either mechanical ventilation or sedation, the simulation procedure was repeated in PSV mode whilst maintaining PSV = 0 cm H_2_O and propofol infusion rate = 0 mcg/kg/min. Progressively increasing ECCO_2_R levels were applied, up to a maximum of 90% of VCO_2_, until LDP targets were met. No titration of ventilation or sedation was applied for this simulated intervention. Patients who did not meet LDP targets at any ECCO_2_R level were deemed to have “treatment failure” with this intervention.

### Simulation variability

To assess the variability of the simulations, the simulation procedure described above was repeated a total of 10 times under both PSV and PAV+. For each simulation, 5000 new patients were generated, with their characteristics determined by random sampling from the predefined distributions (Additional file 1: Table S1). Variability of the simulation was evaluated using a 95% confidence interval for the rate of success in each condition.

### Statistical analysis

Descriptive statistics were reported using mean and standard deviation or median and interquartile range, as appropriate. Proportions are presented as percentages with 95% confidence intervals. The primary outcome was the rate of treatment success in the simulation under PSV in comparison to PAV+ analyzed by logistic regression.

To assess the face validity of the results of the in silico trial, the rate of treatment success in the trial was compared in aggregate to the rate of treatment success reported in a recently published pilot clinical trial of the same LDP titration strategy applied in PSV mode [18]. Because the physiological model requires input variables that are not readily measured in the clinical setting, the simulator could not be directly applied to patients enrolled in the clinical trial. Instead, we derived a statistical model from the in silico trial results and applied this model to the patients in the clinical trial to allow comparison of predicted and observed treatment success in each patient (Fig. 1). This statistical model was derived by identifying the most influential variables determining treatment success in the in silico trial using a random forest model procedure and then selecting the three most influential variables to construct a logistic regression model of treatment success in the in silico trial. This statistical model derived in the in silico trial population was applied in the clinical trial population to predict the probability of treatment success for each patient. Predictive discrimination was quantified by receiver operating characteristic curve analysis.

Finally, a simplified clinical score was derived from a logistic regression model of the in silico trial results by replacing lung compliance (not routinely available without esophageal manometry) with respiratory system elastance. The score was computed from the regression coefficients.

Sample size (“power”) calculation for in silico trials is not limited by usual feasibility concerns. Therefore, we selected a sample size of 5000 patients to permit a sufficient number of treatment success events (assuming a treatment success rate of 40–50%) to have at least 100 events per variable in the random forest model of variable influence, as per previous recommendations [32].

Role of the funding sources: The funding sources cited in the Acknowledgements section had no role in study design, data collection, data analysis, data interpretation, or writing of the report.

## Results

The baseline physiological characteristics of the simulated population are described in Table 1.

**Table 1:** Characteristics of the in silico trial population

		Mode of ventilation
	Patient characteristics [mean (SD)]	PSV or PAV+
Simulated baseline characteristics supplied to the model	Age [years]	55 (10)
	Weight [kg]	85 (15)
	Height [m]	1.70 (0.08)
	Body mass index [kg/m^2^]	29.6 (5.9)
	Sex (female) [%]	49
	PaO_2_ [mmHg]	100 (19)
	VCO_2_ [mL/min]	223 (37)
	Anatomical dead space [mL]	128 (29)
	Alveolar dead space fraction [%]	37 (14)
	Respiratory system resistance [cmH_2_O/(L/s)]	11 (2.4)
	Intrinsic PEEP [cmH_2_O]	1.5 (0.8)
	Lung compliance [mL/cmH_2_O]	47 (13)
	Chest wall compliance [mL/cmH_2_O]	125 (180)
	Strong ion difference [mEq]	34 (7)
	Respiratory rate [/min]	28 (5)

		PSV	PAV+
Simulated baseline patient characteristics computed after applying the model on baseline ventilator and sedation settings	PaCO_2_ [mmHg]	35 (9)	36 (9)
	Plasma bicarbonate [mmol/L]	24 (7)	24 (7)
	pH	7.44 (0.04)	7.43 (0.02)
	Tidal volume (Vt) [mL]	568 (171)	566 (192)
	Tidal volume/kg PBW [mL/kg]	9 (2.9)	9 (3.2)
	Minute ventilation [L/min]	15.6 (4.7)	15.5 (5.1)
	Ventilatory ratio	2.3 (0.9)	2.3 (0.9)
	ΔPes [cmH_2_O]	9 (8)	9 (4)
	ΔP_L,dyn_ [cmH_2_O]	18 (7)	18 (7)

*PSV* pressure support ventilation, *PAV+ * proportional assist ventilation, *BMI* body mass index, *F/M* female/male, *PaO*_*2*_ arterial oxygen pressure, *PaCO*_*2*_ arterial carbon dioxide pressure, *VCO*_*2*_ carbon dioxide production, *Vt* tidal volume, *PBW* predicted body weight, *ΔPes* esophageal pressure swing, *ΔP*_*L,dyn*_ dynamic transpulmonary driving pressure

### Simulated trial outcomes with PSV

**Fig. 2:**
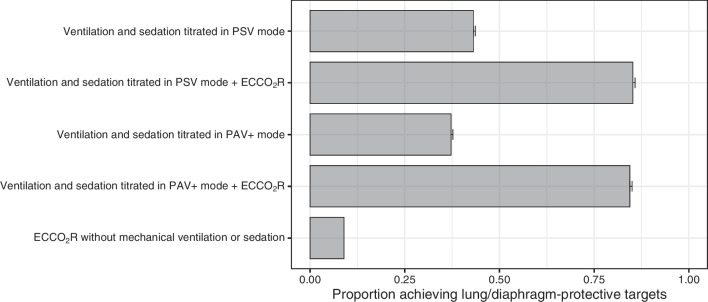
Primary results of the in silico trial. Proportion of the patients achieving LDPV targets at baseline, after applying the ventilation and sedation titration algorithm, and after applying ECCO_2_R if needed in either PSV orPAV+ mode. Error bars indicate 95% confidence intervals for the population proportion

The outcome of the simulated trial in PSV is shown in Fig. 2. At baseline, 754 patients (15%) of the population met the targets of the LDPV (primary success). Modifying sedation and ventilation according to the algorithm enabled an additional 1399 patients (28%) to achieve the LDPV targets, for a total treatment success rate of 43.1% (95% CI 42.6%, 43.6%). Amongst patients who successfully achieved targets, the PSV support level median was 9 cmH_2_O, (IQR 8–10, range 2–10) and treatment success was obtained in over 80% of patients within 3 or fewer cycles of the algorithm.

Combining PSV with adjunctive ECCO_2_R obtained a treatment success rate of 85.2% (95% CI 84.7%, 85.8%) (*p* < 0.0001 for increase in treatment success on ECCO_2_R) (Fig. 2). In patients in whom LDP targets were achieved by the addition of ECCO_2_R, the required ECCO_2_R rate was a median value of 85 ml/min (IQR 50–128) corresponding to 40% (IQR 20–50%) of the VCO_2_.

### Simulated trial outcomes with PAV+

In PAV+ mode on initial settings (50% support), 1565 patients (31%, odds ratio for success under PAV+ vs. PSV at baseline 2.6, 95% CI 2.3–2.8) met LDP targets at baseline (Fig. 2). Modifying sedation and PAV+ support according to the algorithm enabled an additional 293 patients (6%) to achieve LDPV targets, for a total treatment success rate of 37.2% (95% CI 36.7%, 37.7%) (odds ratio for treatment success in comparison to PSV, 0.78, 95% CI 0.73–0.85). Amongst patients who successfully achieved targets, the PAV+ support level was median 0.5, (IQR 0.5–0.5, range 0.3–0.7) and treatment success was obtained in over 80% of patients at baseline.

Combining PAV+ mode with adjunctive ECCO_2_R obtained a treatment success rate of 84.4% (95% CI 83.9%, 85.0%) (*p* < 0.0001 for increase in treatment success with ECCO_2_R) (Fig. 2). In patients in whom LDP targets were achieved by the addition of ECCO_2_R, the final required ECCO_2_R rate was a median value 78 ml/min (IQR 39–124) corresponding to 40% (IQR 20–50%) of the VCO_2_.

### Simulated trial outcomes with ECCO_2_R alone

When applying ECCO_2_R without pressure support or propofol, only 447 patients met LDP targets (9%, odds ratio 0.02, 95% CI 0.015–0.019, for success under ECCO_2_R alone vs ECCO_2_R with PSV + sedation). Only 4 patients met the targets at baseline without any ECCO_2_R assistance.

### Determinants of treatment success in the simulated population

The most influential determinants of treatment success in PSV mode were lung compliance, ventilatory ratio, and strong ion difference (Additional file 1: Figure S2). A multivariable logistic regression model with these three factors (Additional file 1: Table S2) exhibited strong discriminative accuracy for treatment success in PSV mode (C-statistic 0.92).

### Comparing outcomes predicted by the in silico trial to outcomes observed in a clinical trial

**Fig. 3:**
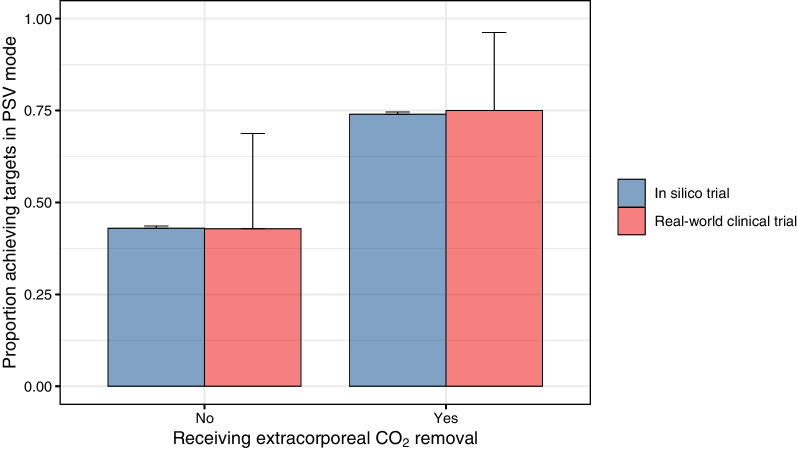
Predicted (in silico trial) and observed (clinical trial) outcomes. The proportion of patients in whom lung- and diaphragm-protective targets were achieved after applying the ventilation and sedation titration algorithm was similar between the in silico trial and a recent clinical trial [18] of a lung- and diaphragm-protective strategy in patients with acute hypoxemic respiratory failure. Error bars indicate 95% confidence intervals for the population proportion

**Fig. 4:**
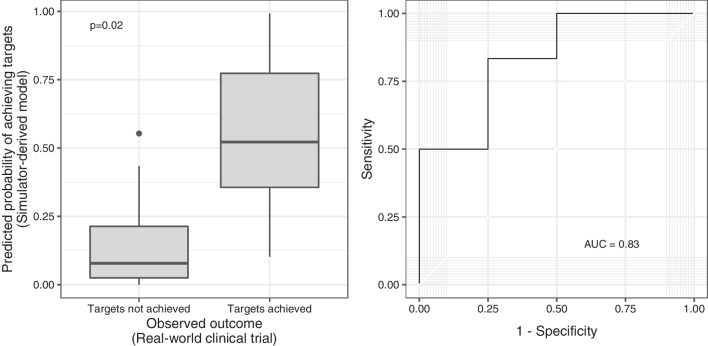
Predicted versus observed treatment success rates in a real-world clinical trial [18]. The predicted probability of success was computed using a three-variable logistic regression model derived from the in silico clinical trial results. The predicted probability of success was significantly greater amongst patients with observed treatment success in comparison to patients with observed treatment failure (*p* = 0.025), and the predicted probability of treatment success discriminated between patients in whom lung- and diaphragm-protective targets were or were not achieved (area under receiver operating characteristic curve = 0.83)

The rates of treatment success in PSV mode without and with adjunctive ECCO_2_R were very similar between the in silico trial and the clinical trial (Fig. 3). The probability of treatment success predicted by the three-variable logistic regression model derived from the in silico trial results was significantly higher in patients in whom LDP targets were successfully achieved in the clinical trial (*p* = 0.02) (Fig. 4). The predicted probability of success discriminated well between patients in whom treatment success was achieved or could not be achieved in the clinical trial (area under receiver operating characteristic curve 0.83, Fig. 4).

### Clinical prediction score to identify patients at high risk of treatment failure

A simplified clinical prediction score (the “ECCO_2_R score”) using readily available clinical variables (ventilatory ratio, respiratory system elastance normalized for predicted body weight) to predict treatment success after titrating ventilation and sedation with or without ECCO_2_R was generated using a logistic regression model fit on the in silico trial results (Additional file 1: Table S2). As described in Additional file 1, the ECCO_2_R score was computed as the sum of the values of normalized respiratory system elastance and ventilatory ratio.

**Fig. 5:**
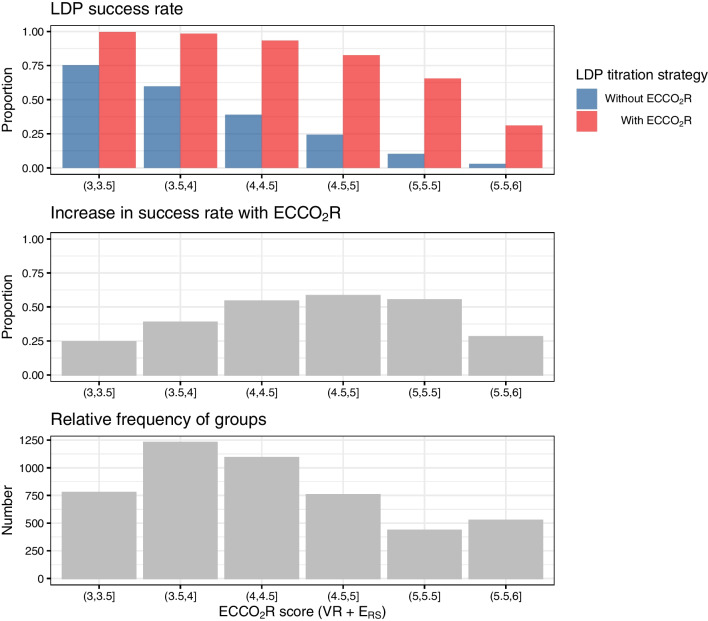
Probability of achieving lung- and diaphragm-protective targets in the in silico trial according to the value of the ECCO_2_R score, a clinical prediction score computed from ventilatory ratio and respiratory system elastance. The relative frequency of each value of the score is shown in the histogram

Simulated rates of treatment success in the in silico trial with PSV with or without adjunctive ECCO_2_R according to the ECCO_2_R score are shown in Fig. 5. The probability of achieving targets by titrating ventilation and sedation in PSV mode progressively decreased with increasing ECCO_2_R score. Adjunctive ECCO_2_R was associated with a relatively small predicted increase in the rate of treatment success at the lowest and highest values of the scores (representing either minimal or extreme physiological derangement, respectively). At intermediate values of the score, use of adjunctive ECCO_2_R was associated with a large increase in the probability of treatment success in the in silico clinical trial.

## Discussion

In this in silico trial of a strategy for titrating ventilator support and sedation to achieve lung- and diaphragm-protective targets, the model predicted that targets would be achieved in ~ 40% of patients in either PSV and PAV+ modes. PAV+ was not associated with a higher rate of success. The rate of achieving LDP targets at baseline was higher in PAV+, whereas PSV achieved a slightly higher success rate after adjusting ventilation and sedation. Adjunctive ECCO_2_R significantly facilitated the success of the LDP strategy but ECCO_2_R alone, on the other hand, had a very low rate of treatment success. In silico trial results corresponded closely to the results obtained in a recent clinical trial of the LDP strategy. The results of this in silico trial require further validation in clinical trials, but the findings in this study provide important insights for patient selection and intervention design for future trials of lung- and diaphragm-protective strategies.

The validity of these in silico trial findings are corroborated by comparison to findings in a recent pilot clinical trial of the same strategy in patients with acute hypoxemic respiratory failure. A regression model for predicting treatment success derived from the in silico data accurately discriminated between treatment success and failure in a “real-world” clinical trial of the ventilation and sedation titration algorithm, and the rates of treatment success in patients treated without or with ECCO_2_R corresponded very closely to the observed results in the clinical trial.

The higher initial success rate in PAV+ suggests that clinicians may find it easier to achieve LDP targets by applying proportional assistance modes like PAV+. In PAV+, the amount of support is proportional to the patient’s inspiratory effort, and this inherently avoids over-assistance to maintain a reasonable level of diaphragm activity [33]. Some studies suggest that allowing the patient’s respiratory control system to determine tidal volume using proportional assistance ventilation will maintain safe tidal volumes, provided respiratory mechanics are not severely deranged [12, 34]. However, similar rates of success are likely to be achieved in the more familiar PSV mode with careful titration of sedation and ventilation.

Previous studies have shown that ECMO or ECCO_2_R is an effective tool to control respiratory drive and effort in patients with acute respiratory failure by reducing ventilatory load [14, 15]. As expected from first principles of physiology, this in silico trial found that applying ECCO_2_R increased the probability of achieving LDP targets; indeed, success was achievable in the vast majority of patients. On the other hand, applying ECCO_2_R without ventilation and sedation was associated with a very low rate of success. It has previously been proposed that respiratory failure might be managed safely with ECCO_2_R alone (so-called ‘awake ECCO_2_R’); our findings suggest that ECCO_2_R alone is unlikely to adequately control respiratory drive and effort or achieve adequate ventilation without mechanical ventilation or sedation, depending on the severity of physiological derangement. During awake ECCO_2_R, the lung-distending pressure is entirely dependent on respiratory effort. When even just 10% of VCO_2_ requires CO_2_ clearance by alveolar ventilation, the tidal volume must exceed the dead space. If elastance or dead space are high, then elevated respiratory effort would be required to achieve that minimal level of alveolar ventilation. Additionally, awake patients with an intact wakefulness drive to breathe would demand a minimum tidal volume to satisfy respiratory drive, even if gas exchange requirements are fully assumed by the extracorporeal circuit [35]. In this case, patients with more severely impaired respiratory mechanics will exhibit substantial respiratory effort and lung-distending pressures, irrespective of extracorporeal CO_2_ clearance.

These data also provide insight as to the strongest determinants of successfully achieving lung- and diaphragm-protective targets: lung compliance, ventilatory ratio (largely a surrogate for pulmonary dead space), and strong ion difference (a measure of metabolic acid–base load). This helps to inform patient selection for adjunctive strategies to facilitate LDP targets: patients with excessive ventilatory demands from any of these sources (mechanics, dead space, metabolic acidosis) are more likely to fail to achieve LDP targets and more likely to require adjunctive interventions to maintain diaphragm activity whilst protecting the lung (e.g. ECCO_2_R, phrenic nerve stimulation). A simple clinical score (the “ECCO_2_R score”) derived from the in silico trial data may help guide patient selection in future trials, although this hypothesis requires prospective confirmation.

### Limitations of our approach

Several limitations should be noted. First, the physiology-based model depends mainly on chemoreceptive (peripheral and central) control of ventilation. The control of ventilation is also influenced by non-chemoreceptive stimuli of ventilatory drive such as reflex and behavioural feedback [19]. Reflex feedback is mostly related to the effect of air flow on the airway and chest wall receptors. Such control mechanisms may be relevant in critically ill patients, but they are not sufficiently characterized to be incorporated in the mathematical model. Not accounting for non-chemoreceptive stimuli of ventilatory drive can lead to some level of inaccuracy of our model. Mathematical models of reflex and behavioural control of respiratory drive are lacking [36].

The relationship between respiratory effort and respiratory rate during mechanical ventilation is complex [17] and the physiology-based model is unable to account for any potential correlation between effort and rate. No empirical data exist to reliably characterize this relationship (to our knowledge), and so we could not specify an a priori basis for computational modelling of the changes in respiratory rate in response to loading conditions. For the purpose of this in silico trial we had to assume a single fixed value of respiratory rate during the simulation. Failing to account for increases in respiratory rate as minute volume requirements increased in the model would have led to overestimation of required tidal volume, lung-distending pressure, and respiratory effort. The available data suggest that respiratory rate is relatively insensitive to changes in respiratory drive and effort [37]. That the clinical trial data corroborated the in silico trial data is somewhat reassuring that the inability to account for respiratory rate variation does not significantly impair model function.

Another limitation of the physiological model is the inability to simulate PEEP and its influence on both mechanics and gas exchange. Recent data suggest that PEEP may have important effects on respiratory effort but this is mediated through the effect of PEEP on dynamic lung compliance [18]. Moreover, in PAV+ mode, PEEP setting is very important. It may heavily influence the true level of assist, especially in patient with a non-linear pressure–volume relationship at the tidal volume range. Adjusting assist without adjusting PEEP could achieve protection but not adequate ventilation. It seems reasonable to say that the use of such a model in a clinical setting would need adjustment for the contribution of PEEP, maybe using dynamic lung compliance as a tool to set up PEEP. Additionally, although the range of values for the simulated patient characteristics are taken from the literature, we did not account for potential correlations amongst these characteristics (i.e. correlation between dead space and mechanics, if any). The similarity of the findings between the in silico trial and the clinical trial provides some reassurance that these limitations do not seriously undermine the validity of the in silico trial results. Finally, it should be emphasized that the exact target range of values for respiratory effort and lung-distending pressure for lung and diaphragm protection have not been validated in clinical trials; the ranges used in this in silico trial represent expert consensus based on available data [11].

## Conclusions

In simulated patients with acute respiratory failure, the success of a lung- and diaphragm-protective ventilation strategy to achieve lung- and diaphragm-protective targets was similar between PSV and PAV+. The addition of adjunctive ECCO_2_R significantly enhanced physiological efficacy. The probability of achieving lung- and diaphragm-protective targets was dependent on lung compliance, ventilatory ratio, and acid–base status.

### Supplementary Information

**Additional file 1**: **Simulation procedure. Table S1.** Range of values for each parameter of the simulated population. **Table S2.** Logistic regression models of treatment success in PSV mode. **Figure S1.** The lung- and diaphragm-protective ventilation and sedatio titration algorithm. **Figure S2.** Relative influence of different patient parameters on the probability of achieving LDP targets during the in silico trial.

## Data Availability

The datasets used and/or analyzed during the current study are available from the corresponding author on reasonable request.
