# Supplementary material for: Lung- and diaphragm-protective strategies in acute respiratory failure: an in silico trial
Source: Intensive Care Med Exp. 2024 Feb 28;12:20. doi: 10.1186/s40635-024-00606-x (PMC10902250; doi:10.1186/s40635-024-00606-x)
Supplement: Supplementary file 1 — Additional file 1: Simulation procedure. Table S1. Range of values for each parameter of the simulated population. Table S2. Logistic regression models of treatment success in PSV mode. Figure S1. The lung- and diaphragm-protective ventilation and sedatio titration algorithm. Figure S2. Relative influence of different patient parameters on the probability of achieving LDP targets during the in silico trial. [file 40635_2024_606_MOESM1_ESM.docx]

**Lung and diaphragm-protective strategies in acute respiratory failure: an *in silico* trial**

Additional file 1

Online Data Supplement

Damian Ratano MD

Binghao Zhang MASc

Jose Dianti MD

Dimitrios Georgopoulos MD PhD

Laurent J Brochard MD

Timothy C Y Chan PhD

Ewan C Goligher MD PhD

**Simulation Procedure**

The process for the simulation was as follows:

1. Generate a random population of 5000 patients using specifications as outlined in **Table S1**.
2. For pressure support ventilation (PSV), generate initial target output variables ΔPes, ΔP_L,dyn_, and pH using the mathematical model of control of breathing (15). Initial values for the controls (pressure support level, ventilator cycling flow threshold, and propofol infusion rate) are specified in **Table S1**.
3. If the initial outputs of a patient meet the LDP targets (ΔP_L,dyn_ <15 cm H_2_O, ΔPes of –3 to –8 cm H_2_O, and a pH >7.25), simulation for that patient was terminated and the patient was classified as “primary success”.
4. For patients whose initial outputs do not meet the LDP targets, a stepwise titration algorithm (17) was applied (**Figure S1**) for a maximum of 20 iterations, with the outputs of the model being evaluated after every iteration. Pressure support level changed in steps of 1 cm H_2_O, with a range of 1 – 15 cm H_2_O. Propofol infusion rate changed in steps of 10 mcg/kg/min, with a range of 20 – 80 mcg/kg/min. Ventilator cycling flow threshold changed in steps of 5% in accordance with the change in pressure support level, with a range of 10% - 50%. If the LDP targets were met at any point, the patient was labelled as “treatment success” and the simulation for that patient was terminated. If LDP targets were not achieved after 20 iterations, the patient was classified as “treatment failure”.
5. For patients who are labelled as “treatment failure”, simulated ECCO_2_R was applied by reducing the rate of CO_2_ production in the model. Afterwards, the titration was performed again, using the same initial control values for a maximum of 10 iterations. Initially, the amount of CO_2_ removed equaled to 10% of the simulated CO_2_ produced. If the LDP targets were unable to be achieved, the amount of CO_2_ removed was increased by 10%, up to a maximum of 90%. If the LDP targets were achieved at any point in this process, the patient was classified as “ECCO_2_R success” and the simulation for that patient was terminated. If the LDP targets were unable to be achieved, the patient was classified as “ECCO_2_R failure”.
6. The process was repeated for proportional assist ventilation (PAV+) with the same patients, with an initial assist level of 0.5. The assist level changed in steps of 0.1, with a range of 0.2 – 0.8.

**Table S1. Range of values for each parameter of the simulated population, mean (SD)**

|  | **Characteristic** | **Specification for Simulation** |
| --- | --- | --- |
| **Management characteristics** | Mode | PSV or PAV |
|  | Pressure support level | 10 cm H_2_O at outset |
|  | Proportional assist ventilation level | 0.5 at outset |
|  | Ventilator cycling flow threshold in PSV | 25% of peak inspiratory flow |
|  | Propofol infusion rate | 20 mcg/kg/min at outset |
| **Patient characteristics** | Area constant | 17.8 (for all) |
|  | PO_2_ at max sensitivity before receptor failure | 30 mm Hg (for all) |
|  | Receptor sensitivity in hyperoxia | 0 L/min/(nM/L) (for all) |
|  | Threshold of central receptors | 31.8 nM/L (for all) |
|  | Threshold of peripheral receptors | 34.6 nM/L (for all) |
|  | PaO_2_ | 100 (20) mm Hg |
|  | Breathing frequency | 28 (5) min^-1^ |
|  | Metabolic production of CO_2_ | 0.22 (0.05) L/min |
|  | Anatomical dead space | 0.125 (0.035) L |
|  | Alveolar dead space fraction | 0.3 (0.2) |
|  | Total respiratory system resistance | 12 (3) cm H_2_O/(L/s) |
|  | Intrinsic PEEP | 1.3 (1) cm H_2_O |
|  | Static respiratory system compliance (normalized by predicted body weight) | 0.0005 (0.0001) (L/kg)/cm H_2_O |
|  | Ratio of lung elastance to total respiratory system elastance | 0.7 (0.1) |
|  | Age | 55 (10) years |
|  | Height | 170 (8) cm |
|  | Sex | 50% female |
|  | Opiate administration with sedation | 50% “yes” |
|  | Plasma phosphate | 0.0004-0.002 mol/L |
|  | Plasma sodium concentration | 0.144 (0.008) mol/L |
|  | Plasma potassium concentration | 0.004 (0.001) mol/L |
|  | Plasma chloride concentration | 0.100 (0.012) mol/L |
|  | Plasma calcium concentration | 0.0021 (0.0003) mol/L |
|  | Plasma magnesium concentration | 0.001 (0.0002) mol/L |
|  | Albumin concentration | 2.7 (0.8) g/dL |

**Table S2. Logistic regression models of treatment success in PSV mode.**

| **Model** | **Parameter** | **Regression coefficient**  **(log odds,**  **mean ± std. error)** | **Coefficient for score*** |
| --- | --- | --- | --- |
| Model for validating *in silico* trial in real patient data  (C-statistic 0·92) | Lung compliance  (L/cm H_2_O) | 131 ± 5  (p<0·0001) | n/a |
|  | Ventilatory ratio  (no units) | –2·9 ± 0·1  (p<0·0001) | n/a |
|  | Strong ion difference (mEq) | 0·28 ± 0·01  (p<0·0001) | n/a |
| Simplified model for clinical prediction | Normalized respiratory system elastance  (cm H_2_O/[mL/kg]) | -1·49 ± 0·06  (p<0·0001) | 1** |
|  | Ventilatory ratio | –2·17 ± 0·10  (p<0·0001) | 1** |

*Clinical prediction score is computed as (normalized respiratory system elastance) + ventilatory ratio. The maximum value is taken to be 6 and the minimum value is taken to be 3.

**Since the coefficients of -1·5 and -2·2 are fairly similar and the distributions of normalized elastance and ventilatory ratio are fairly similar, for the sake of simplicity of clinical application both variables were assigned a coefficient of 1 for the purpose of creating the score.

*
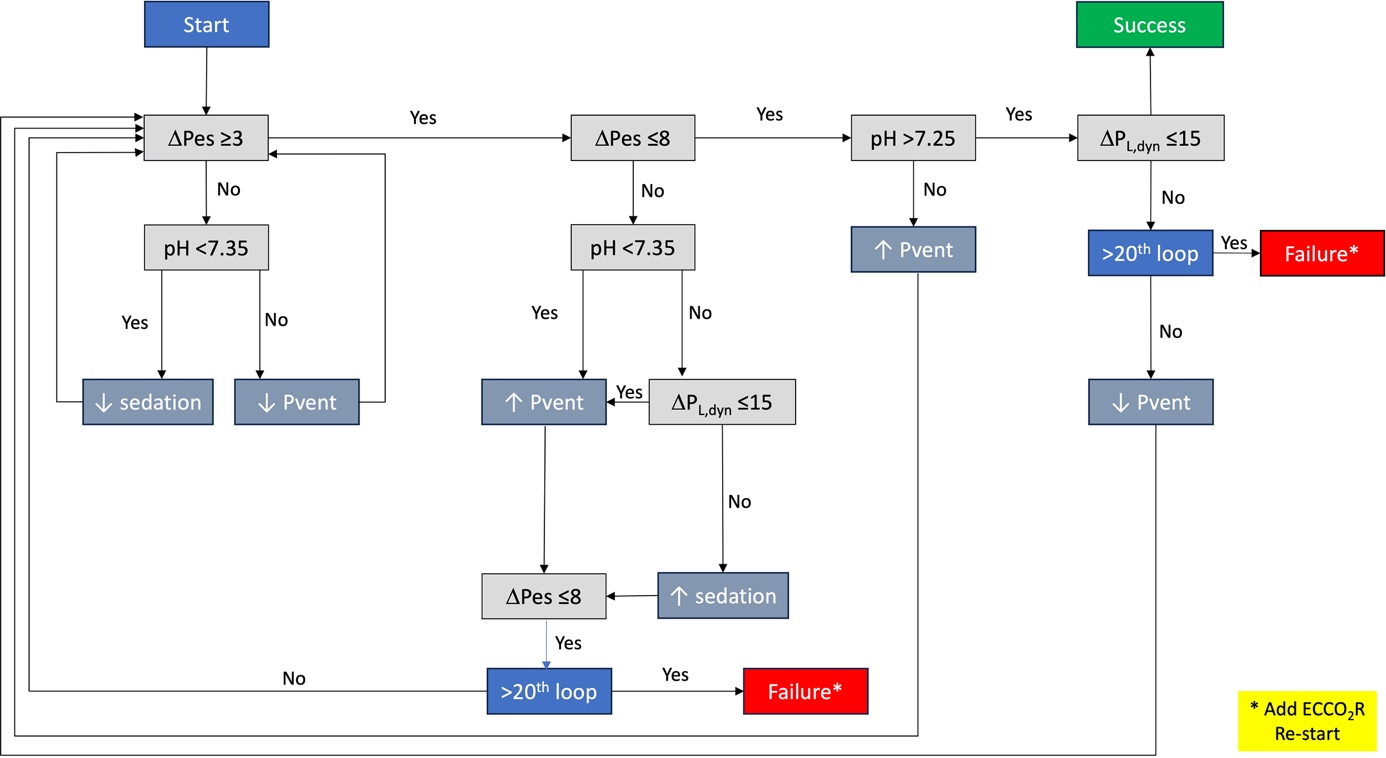
*

**Figure S1.** The lung- and diaphragm-protective ventilation and sedation titration algorithm, adapted from^E1^.

**Figure S2. Relative influence of different patient parameters on the probability of achieving LDP targets during the *in silico* trial.**

**References:**

E1 Dianti J, Fard S, Wong J, Chan TCY, Del Sorbo L, Fan E, et al. Strategies for lung- and diaphragm-protective ventilation in acute hypoxemic respiratory failure: a physiological trial. Crit Care [Internet]. 2022 Aug 29 [cited 2022 Sep 6];26(1):259. Available from: https://ccforum.biomedcentral.com/articles/10.1186/s13054-022-04123-9
